# The effect of strontium ranelate on the healing of a fractured ulna with bone gap in rabbit

**DOI:** 10.1186/s12917-016-0724-6

**Published:** 2016-06-16

**Authors:** Mohd Rafiq Mohd Ibrahim, Simmrat Singh, Azhar Mahmood Merican, Hanumantha Rao Balaji Raghavendran, Malliga Raman Murali, Sangeetha Vasudevaraj Naveen, Tunku Kamarul

**Affiliations:** Tissue Engineering Group (TEG), National Orthopaedic Centre of Excellence in Research and Learning (NOCERAL), Department of Orthopaedic Surgery, Faculty of Medicine, University of Malaya, Kuala Lumpur, Malaysia; Clinical Investigative Centre, Faculty of Medicine, University Malaya Medical Center, Kuala Lumpur, Malaysia

**Keywords:** Fracture, Strontium, Ulna, Osteocalcin, Alkaline phosphatase

## Abstract

**Background:**

Fracture healing in bone gap is one of the major challenges encountered in Orthopedic Surgery. At present, the treatment includes bone graft, employing either internal or external fixation which has a significant impact on the patient, family and even society. New drugs are emerging in the markets such as anabolic bone-forming agents including teriparatide and strontium ranelate to stimulate bone growth. Based on the mechanism of their actions, we embarked on a study on the healing of a fractured ulna with bone gap in a rabbit model. We segregated ten rabbits into two groups: five rabbits in the test group and five rabbits in the control group. We created a 5 mm bone gap in the ulna bone, removing the periosteum as well. Rabbits in the test group received 450 mg/kg of strontium ranelate via oral administration, daily, for six weeks. The x-rays, CT scans and blood tests were performed every two weeks. At the end of six weeks, the rabbits were sacrificed, and the radius and ulna bones harvested for histopathological examination.

**Results:**

Based on the x-rays and CT scans, fracture healing or bone formation was observed to be faster in the control group. From the x-ray findings, 80 % of the fracture united and by CT scan, 60 % of the fracture united in the control group at the end of the six-week study. None of the fractures united in the test group. However, the histopathology report showed that a callus of different stages was being formed in both groups, consisting of 80 % of bone. The serum levels of osteocalcin and alkaline phosphatase initially remained similar up to three weeks and changed slightly at the end of six weeks.

**Conclusions:**

We conclude that the strontium effect begins slowly, and while it may not interfere with bone cell proliferation it may interfere in the mineralization and delay the acute stage of fracture healing. We recommend that a larger sample size and a longer duration of the study period be implemented to confirm our finding.

## Background

The treatment of delayed unions, nonunions, malunions and bone loss pose great challenges for orthopedic surgeons [1]. Fracture healing disorders negatively affect the patient’s quality of life and result in high healthcare costs, as a second surgery is necessitated to stabilize the fracture and stimulate bone biology. In most cases, successful union will be achieved by restoration of the alignment and stable fixation of the fracture. However, many cases call for adjunctive measures such as bone-grafting or bone transports to stimulate bone-healing and fill bone defects. The gold standard for bone defect repairs is autograft but their usage is affected by morbidity at the donor site and limited supply [2]. Limitations of the allograft include the immunogenic response by the host to the graft and the potential for disease transmission. To overcome these limitations an increasing number of new orthobiologic materials are being developed to aid in the management of bony defects. In future, advanced biotechnologies that accelerate fracture healing may be useful tools, which might also prevent the onset of these disorders [3]. The several options available for bone replacement are autologous bone or allogeneic cancellous or cortical bone, demineralized bone matrix, calcium phosphate-based bone-graft substitute or autologous bone marrow. The biology of each of these grafts varies but they perform one or several essential functions such as osteoconduction, osteoinduction, osteogenesis and structural support [4, 5].

Besides bone graft and bone graft substitutes, orthopedic surgeons at present use anabolic agents such as teriparatide and strontium ranelate to promote fracture healing and increase bone quality. An anabolic agent is one that raises the bone strength by increasing the bone mass due to an overall increase in bone remodeling combined with a positive bone balance [6]. Several case reports on strontium promoting fracture healing have been reported in the literature. Strontium ranelate accelerates filling of the bone defect and improves the properties of bone healing in rats [7]. Strontium nucleus is almost similar in size to that of calcium, the body easily absorbs it, incorporating it into the bones and tooth enamel instead of calcium [8]. Strontium ranelate acts via a dual action mode with its bone-forming and bone-resorbing properties, resulting in improved bone microarchitecture [9]. This agent is approved for the prevention of vertebral and hip fracture in postmenopausal osteoporosis. Preclinical studies have demonstrated enhanced fracture healing and osseo-integration with strontium ranelate, coupled with the improvement in bone microarchitecture [9]. Studies have also showed contradicting results in which 3 weeks of treatment with strotium increased the thickness of the healing mid-femoral cortical bone defects in rats, but did not influence volume, mechanical properties, periosteal callus volume, either 3 or 8 weeks. Furthermore, strontium had no effect on the microstructure and mechanical properties of the vertebral bodies [10, 11].

The ulna is found to have similar function, in both humans and four-footed animals. If the ulna breaks, it will most commonly occur at either the point where the radius and ulna form a joint or where the ulna forms a joint with the hand's carpal bones. Ulnar fractures cause severe pain, difficulty in moving the affected joint and even lead to deformity of the arm if the fracture is compound. So far reported studies have focused on the development of biocomposite materials or scaffold on different type of bone fractures and as well as ulna bone, while the effect of strontium ranelate has not been tested on ulna bone. The maximum period for spontaneous healing of the bone fracture is around 6 weeks, so we anticipated the treatment with strontium ranelate would trigger faster healing in rabbits compared with control [12].

## Methods

### Design and methodology

In this study, New Zealand white male rabbits (*Oryctolagus cuniculus; n* = 5) were used. Prior to this study, approval was obtained from the animal ethics committee of the Animal House of the University Malaya Medical Centre (UMMC). All the rabbits were maintained in accordance with the Institutional Animal Care & Use Committee approved protocol.

The rabbits selected for the study were all males, between 28 and 32 weeks of age, and within the weight range of 2.0–2.5 kg at the time of the surgery. All of the rabbits had free access to pellet food and water *ad libitum* and were housed in the animal experimental block of the UMMC at a constant temperature of 26–28 °C and a humidity of 60 %. The rabbits were caged individually in stainless steel cages measuring 9 cubic meters, employing a fenestrated flooring to allow the feces or droppings to fall into a collection pan.

### Sterilization

All the instruments and gauze employed in the surgical procedure were sterilized by autoclaving in the Aesculap 420 laboratory and Eschmann SES 225B autoclave. The instruments were first washed under running water with disinfectant soap and sterilized at a pressure of 220 kPa and a temperature of 134 °C for approximately 15 min. Then they were automatically dried by the autoclaving machine and left for one hour to cool down.

All the surgical procedures were performed in the Animal Theater in the Animal House. Marking the ears of all the rabbits was done with a number assigned to them using a permanent marker for identification. All the cages were also labeled according to their respective numbers. The animals were weighed prior to the surgical procedure, and the amount of anesthesia (Ketamine 20 mg/kg IM) was calculated including the sedative and muscle relaxant, Xylazine (3 mg/kg). Then the antibiotic (Kombitrim 240, 1 ml/10 kg IM) was injected intra-muscularly. The left fore-leg of the rabbit was shaved. All the rabbits gained 0.1–0.3 kg on average throughout the experiment.

### Surgical techniques

After anesthesia had been achieved and the limb shaved, the rabbit then was placed on the operating table in the supine position. The left fore-leg was then cleaned with povidone and properly draped with disposable sterile drapes. The surgery was then performed using the aseptic technique taking adequate precautions all the time.

The center of the ulna bone at the diaphysis was identified and marked on the skin surface. Local anesthesia was administered to the rabbit at the planned incision site. Next, 1.5 cm long longitudinal incisions were made. The intramuscular septum was identified and the mid-shaft of the ulna was exposed. Soft tissues were gently dissected to expose the bone. A longitudinal incision 4 cm in length was made on the anteromedial surface of the left ulna at the cutaneous border, 2 cm proximal and 2 cm distal to the center of the diaphysis which had been marked prior. The fascia was then cut and the extensor muscles were identified and retracted to expose the ulna bone. Care was taken to split the muscular layer by blunt dissection to expose the ulnar bone. The planned site for the osteotomy was marked on the bone, and measured with a small stainless steel ruler. The osteotomy was then performed using an oscillating saw, carefully avoiding damage to the periosteum. Washing was done with some normal saline during the osteotomy. A 5 mm defect was created in each ulna. All the rabbits tolerated the surgery well and resumed their normal activity within a few days. No wound infection was observed. All the rabbits were freely ambulating one day post-surgery.

No neurological insults such as paralysis, convulsion, respiratory distress or signs of pain were observed.

### Strontium treatment

Each rabbit was supplemented (orally) with 5 ml of strontium ranelate solution (2 grams of strontium ranelate mixed in 10 ml of distilled water) in the treatment group using a feeding tube for six weeks.

### Macroscopic evaluation

After necropsy, the radius and ulna were harvested and the soft tissue removed. The bone defect site was observed macroscopically and photographs were also recorded.

### X-ray and CT scan

The bone defect healing of all the rabbits was evaluated using the X-ray (DRX-Evolution, 50kVp, 2mAs Carestream, Malaysia) and CT scan. Scanning was done at weeks 1, 3 and 6 for each rabbit. They were scanned weekly for six weeks to determine the progress of defect healing in each group. While under general anesthesia, the rabbits were positioned accurately on the CT scan machine (Siemens Somatom Definition AS 128, Germany) in the right lateral position with the left fore-leg elevated on a sample holder. The long axes of the radius and ulna were aligned orthogonally to the axis of the x-ray beam. All the radius and ulnar bones were scanned using 35.5 μm isotropic resolutions at 80 kVp energy and 60 μA intensity in 150 ms integration time. Images had 0.6 mm slice thickness and resolution of 2048 × 2048 pixels. Images obtained from the scan were saved in DICOM (Digital Imaging and Communication in Medicine) format. The assessment such as calcification, appearance of callus, continuity of bone trabeculae was done by a Senior Orthopedic Surgeon who was blinded using the same score.

### Qualitative bone growth evaluation

The radiographs were evaluated objectively and subjectively in collaboration with a consultant orthopedic surgeon. They were evaluated for initial and bridging callus formation. The 3D reconstruction images were obtained for better viewing and bone regeneration evaluation. For this study, we defined union based on the radiographic evaluations. Union was considered to have been achieved in the rabbit ulnar model if visible bridging callus was observed across the fracture on the radiographs, which was grade 4 and above on the Cheung (2000) criteria. Bridging callus needed to be visible in three cortices on two views on the x-ray. We could not do a clinical assessment for the fracture union because the radius was intact and the rabbits were able to weight-bear immediately post -surgery. The pattern and behavior of the bone growth in each group was also evaluated and analyzed from the radiographs enlisting the help of a radiologist.

### Biochemical test

Serum levels of osteocalcin, ALP, calcium and phosphate at weeks 1, 3 and 6 were obtained via the ear vein and centrifuged at 10,000 x g for 10 min. The plasma was then analyzed for osteocalcin, ALP, calcium and phosphate concentrations using the corresponding kit according to the Standard Operating Procedure in the Tissue Engineering Group Laboratory in the National Orthopedic Center for Excellence and Learning (NORCERAL), University of Malaya. The results were measured with the respective optical densities for each parameter on a microplate reader.

### Histopathology

At the culmination of the study after necropsy, the radius and ulnar bones were harvested and the specimens prepared for histopathological slides. Samples for histological evaluation were stored in 10 % neutral buffered formalin for four days and then decalcified for five days in 10 % formic acid. The samples thus processed were embedded in paraffin, sectioned longitudinally and stained with hematoxylin-eosin (Sigma USA). The sections were examined under a light microscope with magnification. The histopathological sections were examined by a single pathologist who was also blinded regarding the content of the current study. The progression of the fracture callus in each specimen was quantified based on the relative percentages of the lamellar bone, woven bone, cartilage, fibrous tissues, endochondral ossification and intramembranous ossification. The qualitative bone growth data were collected using x-ray and CT scan. Statistical analysis was done using the Fisher Exact Test.

## Results

**Fig. 1:**
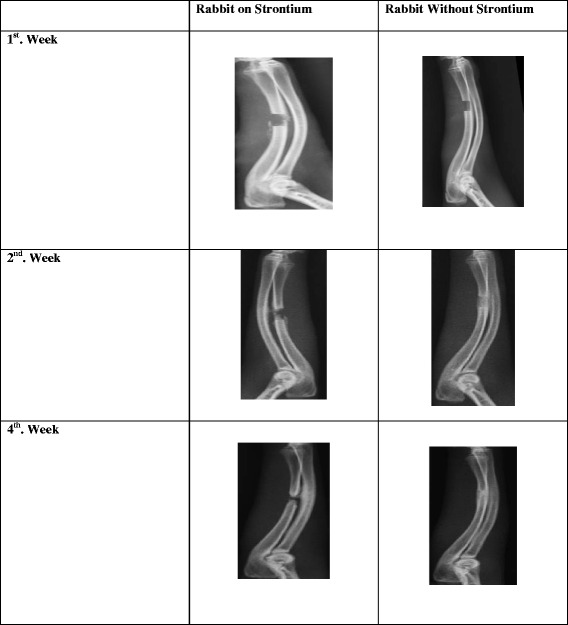
Comparison of radius and ulna radiographs of rabbits with and without Strontium ranelate. The pattern of callus formation in the group treated with strontium or without strontium was observed using radiographs

**Fig. 2:**
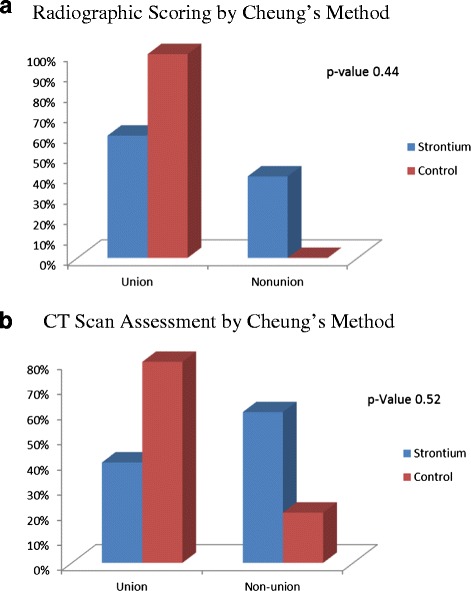
**a** Radiographic scoring by Cheung’s method and **b** CT Scan Assessment by Cheung’s Method, Based on the radio graphical data X-Ray and CT scan the percentage of healing has been indicated

Radiographic examination of the radius-ulna of the strontium treated rabbits was performed at the end of study. None of the samples showed radiological union at the end of the six-week study (Fig. 1). However, excessive callus formation was observed in between the radius and ulna rather than in the fracture gap. From the findings mentioned above it was evident that the strontium treatment did not significantly improve fracture healing in rabbit ulna with bone gap. The union and nonunion rates using x-ray in the test and control groups were assessed by Cheung’s method (Fig. 2a & b) and the *p*-value was calculated as 0.44 using Fisher Exact Test. There is no association of healing of the ulnar fracture with strontium.

An obvious increase in the union rate was recorded using Cheung’s scoring method. However, the control group showed a better union rate on scoring method. All the rabbits were reviewed and the CT scans were taken within the first week post-surgery. No evidence of any callus formation was observed and the 0.5 cm gap was clearly seen. At around three weeks, evidence of minimal callus formation was seen. The callus began to form from the edges of the osteotomized bones. More calluses were noted in between the radius and ulna rather than within the bone gap. At six weeks, only two samples showed radiological union.

**Fig. 3:**
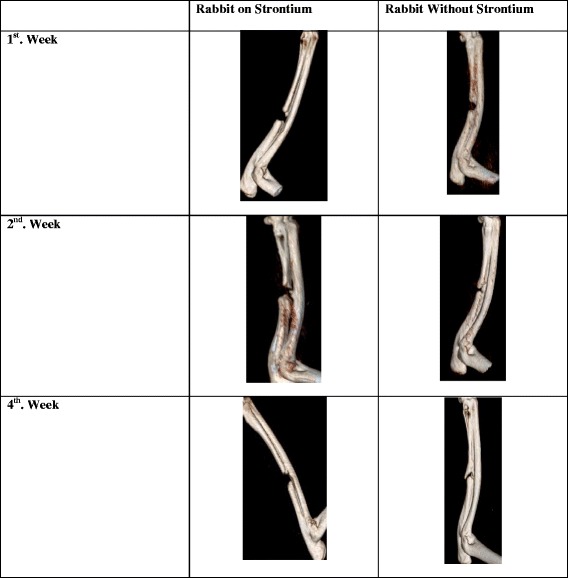
Comparison of radius and ulna CT scans of rabbits with and without Strontium ranelate. The pattern of bone repair in the group treated with strontium or without strontium was observed using radiographs

**Fig. 4:**
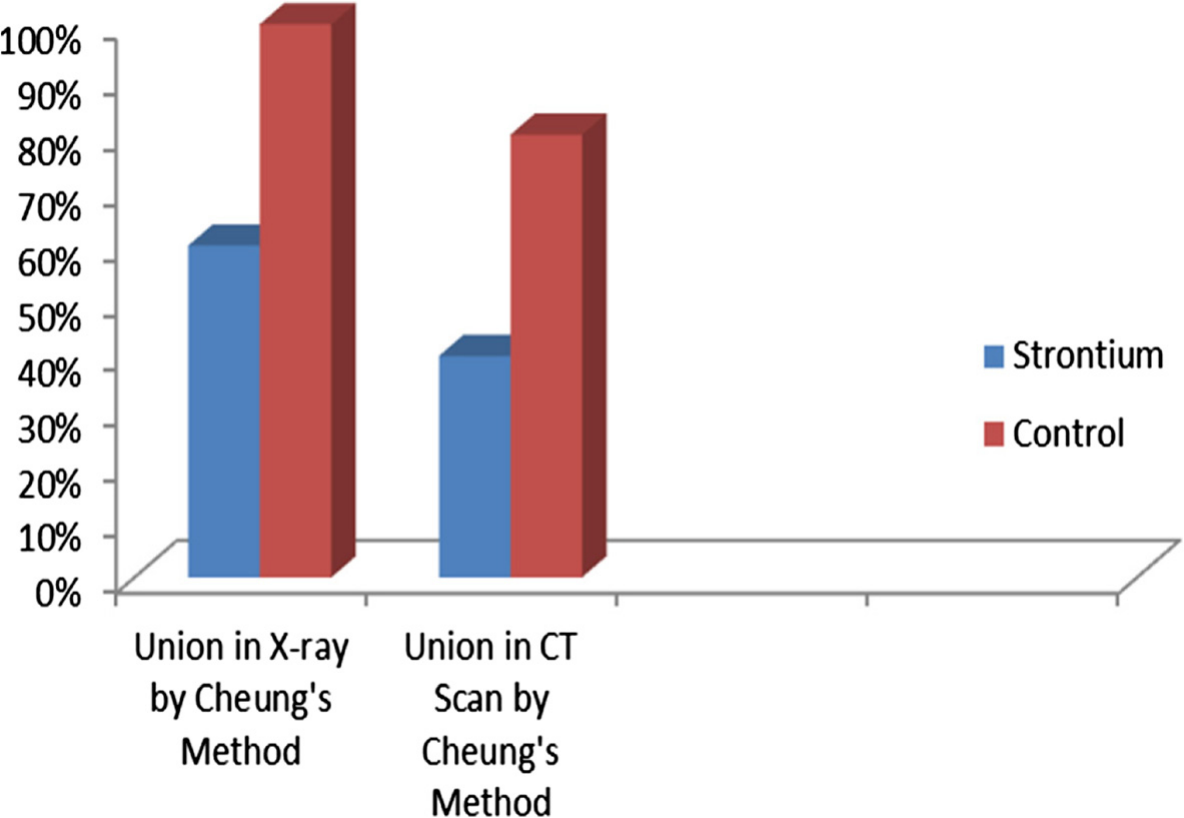
Comparison showing X-ray and CT scan by Cheung’s scoring system. The healing percentage has been indicated in scale based on the callus formation

The CT scans of the rabbit fore-arm at the end of study in Group A (with strontium) and Group B (without Strontium) at the end of study (without strontium) are shown in Fig. 3. Post-surgery all the rabbits in both groups were reviewed and the CT scans were done within the first week of surgery. No evidence of any callus formation was initially found. At three weeks post-surgery, patchy calcifications were noted around the center of the defect and bone edges. The patchy calcifications moved towards the center of the bone defect. The callus in the center of the defect enlarged gradually and expanded proximally and distally. At around four weeks post-surgery, the proximal callus, which grew faster, met and united with the center callus. Then, it slowly grew distally and met the distal callus at around six weeks. At this point in time, no fracture gaps were observed. The bone growths were noted to fill up the whole osteotomy site. At six weeks, the fracture had united and there were continuity between the bone trabeculae but no remodeling noted. The diameter sizes of the newly formed bone were smaller than the actual defect. The *p*-value by Fisher Exact Test is 0.52. In the control group, the union rate is 100 % with x-ray and 80 % with the CT scan using Cheung’s scoring methodology, which is almost two times higher than that of the test group. A comparison of the union rates from the x-rays and CT scans by Cheung’s Method are shown in Fig. 4.

**Fig. 5:**
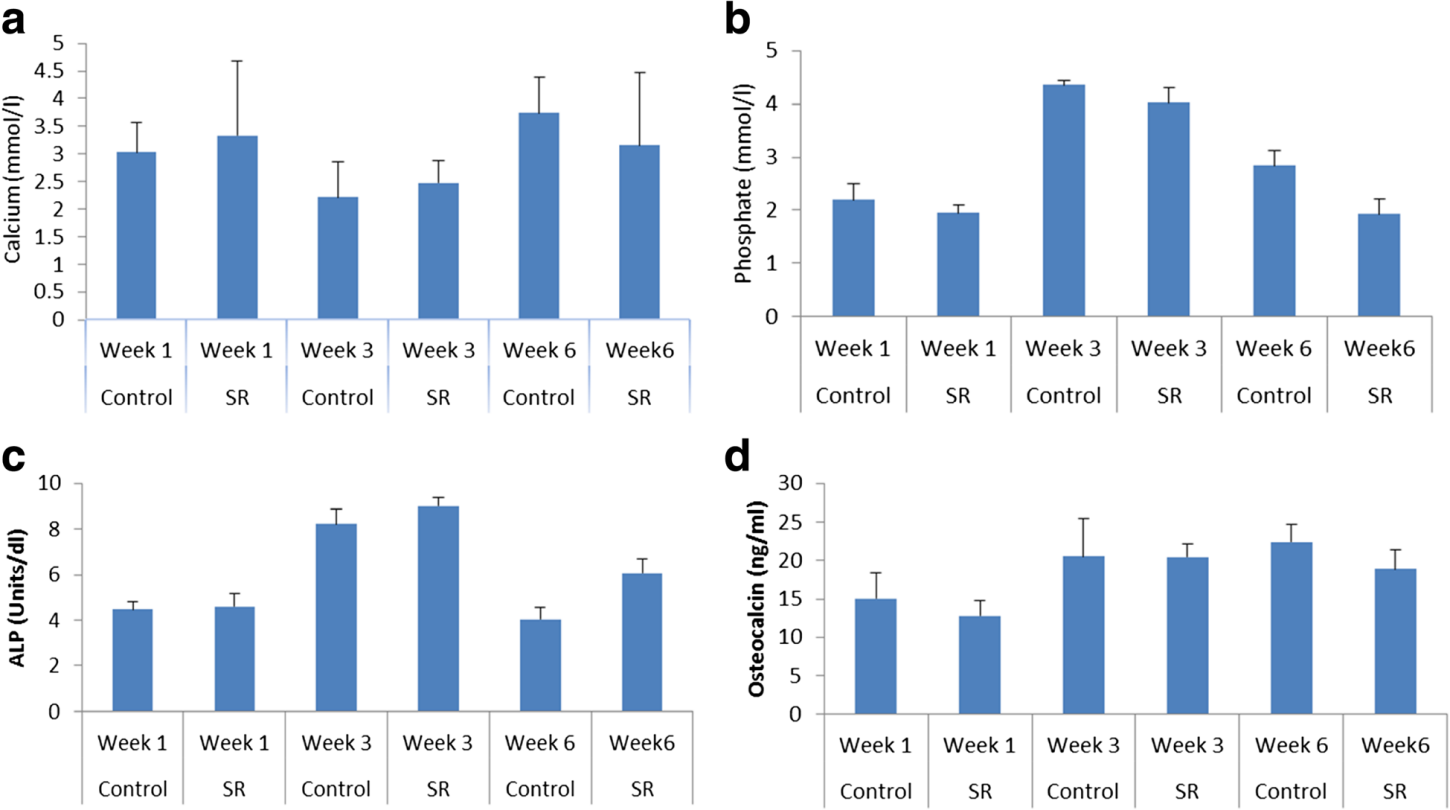
**a**-**d** The levels of calcium, phosphate, ALP, and osteocalcin release in serum at variable time points. Serum separated from blood samples were examined using microtitre plate reader

The biochemical markers including the levels of serum alkaline phosphatase, serum calcium, serum phosphate and serum osteocalcin were taken at weeks 1, 3 and 6 post surgery (Figs. 5a-d). The level serum markers were compared as the callus grew old. Serum levels of calcium and phosphate showed no change at weeks 1, 3 and 6. However, a significant statistical difference was observed in the serum levels of ALP and osteocalcin at week six, where the serum ALP was higher and osteocalcin was lower in the strontium treated group. Based on the histological examination, the grading and percentage of lamellar bone, woven bone, cartilage and fibrous tissue are shown in (Tables 1 & 2). The presence of lamellar bone in the specimen with radiological nonunion may have been contributed by the radius. However, no significant difference in the healing was observed between the strontium treated and untreated groups.

**Table 1:** A six-point score to grade the radiographs

Grade	Radiographic description
Grade 1	Indicates no calcification at the fracture site.
Grade 2	Indicates patchy calcification
Grade 3	Indicates that the calcification takes on the appearance of the callus
Grade 4	Shows callus bridging across the fracture gap
Grade 5	Indicates continuity of bone trabeculae
Grade 6	Demonstrates remodeling to normal bone.

**Table 2:** Histopathological examination and statistical analysis

	Lamellar bone	Woven bone	Cartilage	Fibrous tissue
Control group	70 %	20 %	5 %	4 %
Test group	58 %	28 %	7 %	7 %
*p*-value	0.53	0.64	0.17	0.30

## Discussion

Fracture healing in the presence of bone defect is a significant clinical issue. Non-union is a well-known complication of segmental loss in bone defect [10]. One of the main challenges in successful bone healing and new bone formation is the in-growth of soft tissue which may disturb the osteogenesis and cause non-union. Such defects greatly impact the patient’s quality of life due to limb length discrepancy and prolonged postoperative treatment. Furthermore, it will become an added financial load to the patient causing a socio-economic burden to the family as treatments for this condition are complicated. The current treatment option involves the use of the Ilizarov technique by distraction osteogenesis [11]. However, this treatment includes complications of its own; pin site infection, bulky implant, expensive cost, long hospital stay etc. Recently, anabolic bone-forming agents on fractures in animal models have been reported. The ulnar bone was selected in this study, as it is one of the common sites for fracture and segmental bone loss in humans, especially in trauma cases. Rabbit ulnar bone is easily accessible and the bone size is also appropriate to use. It is relatively easier to perform the surgical procedure in the ulnar bone as it is subcutaneous in location with less soft tissue coverage. Furthermore, it has a splinting effect from the radius and no implant is required.

A 0.5 cm bone defect was created in this study as we desired to see the difference in the physiological repair process through the fracture gap between the group undergoing strontium ranelate treatment and the control group. The ulnar diameter on average was 6–8 mm, measured at the midshaft region. The 0.5 cm bone defects were made in the rabbits at the midshaft of the bone to assess the osteogenesis capacity of the rabbit ulna through the fracture gap. According to the earlier report, in the short experimental time, bone healing was partial and involved the immature bone and fibrous connective tissues [13]. Under such conditions, it became possible to evaluate the effect of an agent in the healing process and whether the healing would be satisfactory as far as tissue quantity and quality were concerned. The callus formation in relation to the repair period was required to assess the healing bone quantitatively and qualitatively; however, the treatment was not significant as observed in the group given strontium ranelate when compared with the control group [14]. In fact, the best tissue quality should be achieved within the shortest time period, assuming that the bone will be remodelled during the animal’s entire lifetime. In general, bone regeneration will be initiated from the periosteum (periosteal callus) and grow in a circumferential pattern from the outside into the centre of the defect. Bone regeneration without periosteum will occur from the primary response callus initially, later the medullary callus and follow a linear growth pattern of growth. The proximal bone edge callus will grow faster than the distal bone edge callus. In our study, the periosteum was removed in both the control group and test group which will exert a negative effect on bone healing. However, in the control group, the fracture unites even without the periosteum when compared with the test group, which received the bone stimulant, strontium ranelate. Therefore, we may conclude that strontium may not be effective if the periosteum is removed. Studies have previously demonstrated that periosteum is important for the acceleration of bone growth after stripping. The grown acceleration has been reported after transection of periosteal and resection [15–17]. Reports has also compared the different procedures head to head in the same mammalian model, transverse transection of the longitudinal periosteal fibers appears to be the most important aspect in accelerating growth [18–20].

From the X-ray findings, four of the five samples in the control group fulfilled the criteria for union using cheung’s scoring method, all five samples from the control group and three samples from the strontium group showed bony union. At the end of the six-week study, four samples from the control group and two samples from the strontium group achieved radiological union from the CT scan findings using Cheung’s method. The radiological findings revealed an association between the use of strontium ranelate and delayed unions or non-unions. Fibrous tissues (fibroblast) which grow faster than the bone (osteoblast) were observed to rapidly migrate and repopulate into the bone defect; however, no significant effect was observed after six weeks between the strontium ranelate treated group and the untreated group [21].

All the samples showed 80 % bone formation within the fracture gap created. The bones that formed were either lamellar bone or bone woven with the minimal fibrous tissue formation, between 5 and 10 %. We expected to see more fibrous tissues in the specimens from the radiological findings of the big bone gaps. Instead, we observed various stages of callus formation. The seven slides were prepared initially by cutting the radius and ulna longitudinally in the sagittal plane to facilitate the comparison of the callus and surrounding normal bones. When the results showed that 80 % of the bones in all the slides were in contrast to our imaging studies, we suspected a mixing of the callus with the surrounding normal bones. We removed the radius in the last three slides prepared, but we still observed the same results. These results demonstrated that the strontium ranelate treatment could interfere with the mineralization process, even though it did not inhibit callus formation. The results of the blood parameters showed no significant statistical difference in the serum levels of calcium and phosphate at weeks 1, 3 and 6. Statistical difference was noted in the serum alkaline phosphatase and osteocalcin at week six with the serum ALP being higher and serum osteocalcin dropping lower in the strontium treated group. This shows that strontium has slow action onset on the bone markers.

Reports have shown strontium ranelate has bone anabolic and anti-resorption effect which probably increase healing of bone defects [22–24]. In addition some reports have shown strontium ranelate seems to decrease bone resorption as well preserves or increases bone formation. Preclinical studies on fracture healing have shown that it has a positive effect on hard callus formation and bone strength [25, 26]. From our assessment using the radiographs and CT scans, strontium ranelate does not promote fracture healing within the first six weeks, especially a fracture with a bone gap, as none of the fractures were observed to unite in the treatment group. However, the HPE report revealed that the gap became filled with lamellar bone or woven bone of differing maturity. It may have initially delayed the fracture healing or impaired the mineralization process. However, changes in the serum levels of the bone markers such as osteocalcin and alkaline phosphatase showed that strontium has a slow onset of action on bone activity. We are, however, still unsure about the long-term effect of the strontium on fracture healing with bone gap. However, based on the x-ray and CT scan findings, strontium did not have a significant impact on fracture healing in the acute setting compared with the control group. One of the reasons why the callus was unable to bridge the gap could be the absence of the perisoteum. The surrounding soft tissue could have prolapsed and blocked the bone gap thus inhibiting the osteogenesis process. But, in specimens with the strontium supplement, the bone grew faster in any case. One of the possibilities is that the periosteum is essential for the strontium to act.

## Conclusions

In conclusion, strontium ranelate is not found to promote bone healing and in fact may delay bone formation, especially during the early stages of fracture healing with bone gap, in rabbit. Further studies are warranted using bigger animal models to confirm the role of strontium ranelate.
